# Isolated pan‐pontine posterior reversible encephalopathy syndrome in a patient with uncontrolled hypertension

**DOI:** 10.1002/ccr3.1888

**Published:** 2018-11-11

**Authors:** Jacqueline Marie Gowan, Antonio Liu

**Affiliations:** ^1^ Department of Neurology California Hospital Medical Center Los Angeles California

**Keywords:** albuminocytologic CSF, central variant PRES, encephalopathy, hypertension, MRI, pan‐pontine

## Abstract

Recognition of central variant PRES is key to avoiding detrimental treatment. The pons may be the most vulnerable area in central PRES, and delayed presentation may result in greater damage. CSF reporting may lead to eventual recognition of a common profile, and possible aid in future diagnosis.

## 1 INTRODUCTION

Posterior reversible encephalopathy syndrome (PRES) has been described as a clinicoradiologic syndrome, with the majority of MRI findings consisting of symmetric edematous changes to theparietal‐occipital areas of the cortex and subcortical white matter.^1^ Patients typically present with vague symptoms such as headache, vision changes, paresis, nausea, altered mental status, and in few cases seizures.^1^ PRES is typically brought on secondarily to a variety of processes such as uncontrolled hypertension, immunosuppressive medications, eclampsia/pre‐eclampsia, uremia caused by renal failure, antineoplastic medications, lupus, or sepsis^2^. Symptoms and radiographic evidence of PRES have shown to improve greatly once the primary disturbance is under control.

Although the exact mechanism of the pathogenesis of PRES remains uncertain, failure of cerebral autoregulation during times of fluctuating blood pressure, as well as endothelial dysfunction of the blood‐brain barrier (BBB) are believed to play a part.^3^, ^4^ Breakdown of the BBB allows for hematogenous products to diffuse slowly over time into the parenchyma of the brain, resulting in areas of edema and dysfunction.^4^, ^5^

The most commonly affected regions in PRES are the parietal and occipital regions of the cortex, followed by the frontal lobes, the inferior temporal junction, and the cerebellum.^1^ Deemed “Classical PRES”, over 90% of patients with PRES have some form of parietal, occipital, and/or deep subcortical involvement.^1^, ^2^, ^3^ The lack of sympathetic innervation to these areas, and thus the decreased ability to regulate perfusion during times of increased blood pressure has been proposed as a possibility as to why these regions are so often affected.^3^

In roughly 4% of cases however, as demonstrated by McKinney et al in 2013, an atypical variant of PRES (dubbed central variant PRES or hypertensive brainstem encephalopathy) exists, in which the parietal‐occipital regions, as well as the subcortical white matter are spared. Instead, edematous changes are found throughout the basal ganglia, thalami, cerebellum, and pons.^2^ The McKinney study documented 124 patients with PRES and found only five to have exclusive involvement of these central structures with preservation of the cortical regions normally seen in classical PRES.^2^ All of the patients with central variant PRES had hypertension at the time of presentation, two were additionally taking cyclosporine, and one was pre‐eclamptic.

Our case involves a 47‐year‐old woman with a history of uncontrolled hypertension, diabetes and end‐stage renal disease, who presented for episodes of waxing and waning confusion, unbalanced gait, and fatigue following a ground level fall 1 week prior. The patient's family reported she had been trying to get control of her blood pressure for years, but had yet to find the right combination of medications and worried she would soon have to resort to hemodialysis.

## 2 CASE REPORT

Our patient, a 47‐year‐old woman with a medical history of hypertension, type II diabetes mellitus, and end‐stage renal disease, presented with her family to the emergency department (ED) with worsening episodes of confusion, unsteady gait, and fatigue, after an episode of syncope and ground level fall 1 week prior. Glasgow coma scale (GCS) was determined to be 13 on her arrival, and work‐up for stroke was initiated. CT scan of the head, as well as ECHO of the carotids, and ECG were all unremarkable. Of note, the patient's blood pressure on admission was 180/99 mm Hg. Additionally, demonstrating how poor the patient's kidney function was at that time, she was noted to have a creatinine of 3.8, a BUN of 52, and glomerular filtration rate of 13. The patient's family were insistent that patient had been adherent with her blood pressure medications at home but had been having issues controlling her it for years with medical management alone. Her home regimen consisted of Furosemide 40 mg BID, Hydralazine 100 mg TID, and Nifedipine 60 mg extended release daily.

On examination, the patient was somnolent and confused. Orientated only to herself, but able to follow simple commands such as “squeeze my hand” and “raise your right arm”. Pupils were equal and reactive to light. Patient showed no focal neurological deficits either on the face or any of her extremities. No asterixis was observed, reflexes were 2 + throughout, and plantar reflexes were down turning on both sides. Heart and breath sounds were normal. Patient's family admitted she was having trouble keeping her balance while ambulating around the time of the fall, and even more so thereafter. Due to the patient's somnolence during the time of the encounter, gait examination was deferred.

**Figure 1:**
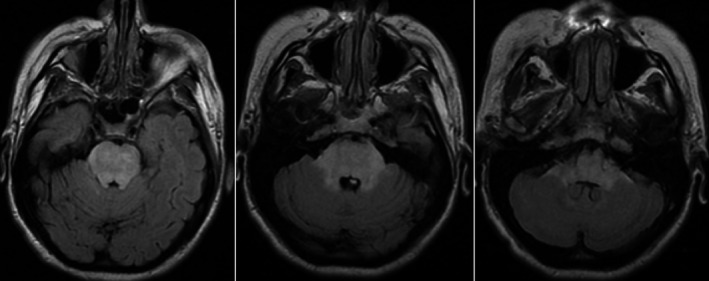
FLAIR MRI on day 1 of admission. Note hyperintense signaling in the entirety of the pons, and partial cerebellum

MRI of the brain showed a much more serious picture of the patient's condition in comparison with the benign results the CT scan had yielded in the ED (Figure 1). The entirety of the pons demonstrated hyperintense signaling on FLAIR MRI imaging indicative of edematous change, which was also found in partial areas of the midbrain, cerebellum, and deep white matter. Diffusion weighted imaging did not show increased signaling, ruling out an acute infarct. Lumbar puncture returned no leukocytes, a glucose of 30, a slightly higher than normal protein of 46, albumin of 22, PCR negative for common viruses (HSV, West Nile, or Hepatitis Viruses), and did not gram stain. Differentials initially included uremic encephalopathy, pontine myelinolysis, and PRES.

During the first two days of hospital admission, the patient's blood pressure fluctuated greatly from a systolic pressure between 106 and 187 mm Hg, and a diastolic between 58 and 96 mm Hg. Medications such as valsartan, carvedilol, and metoprolol were added to the patient's regimen, all in an attempt to take control of her blood pressure. Once it became clear that medicine alone would be unable to control her blood pressure, the decision was made to begin hemodialysis on hospital day 2 in order to curb it more aggressively, as the patient's clinical presentation was not improving. Furthermore, the patient's creatinine and BUN had continued to climb (4.7 and 55, respectively), which was an additional factor in favor of hemodialysis.

**Figure 2:**
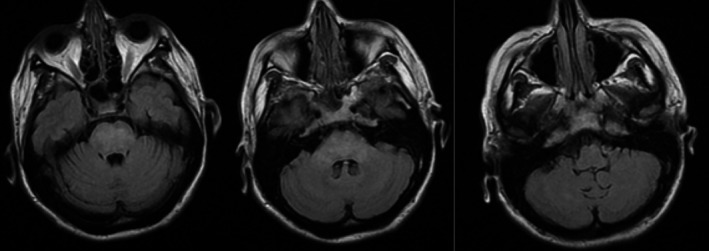
FLAIR MRI on day 9 of admission. Note improvement of hyperintense signaling within the pons in comparison with hospital day 1

On hospital day 4, as her blood pressure slowly lowered to an acceptable level (<140/90), the patient's family noted a marked improvement in her alertness, GCS rose from 13 to 15, and the patient regained the ability to converse appropriately. FLAIR MRI of the brain on hospital day 4 showed improvement of affected hyperintense areas compared to images taken on hospital day 1, and even more so on hospital day 9 (Figure 2). The decision to repeat the MRI on hospital day 9 was both for confirmation of PRES, as well as for academic purposes. The patient was discharged with a blood pressure of 127/66 mmHg, a creatinine of 2.6, and a BUN of 18. Outpatient hemodialysis was arranged for both blood pressure control, as well as for kidney failure.

## 3 DISCUSSION

It is important to continue to highlight cases of central variant PRES, as this particular variant is so rare and misdiagnosis on MRI can lead to delay in appropriate treatment, or in the worst case, implementation of detrimental treatment.MRI imaging of central variant PRES mimics rhombencephalitis, viral encephalitis, pontine myelinolysis, or even an infarct to name a few.^2^, ^3^ For example, if PRES was to be misdiagnosed as an ischemic infarct, the allowance of permissive hypertension may exacerbate parenchyma edema even further. Reporting cases of central variant PRES is therefore important, as it brings to light this presentation, in the hopes of it becoming common medical knowledge among radiologists and neurologists alike.

Furthermore, because our patient had a history of CKD (as many others suffering from PRES do) the question was raised as to whether or not she was in fact suffering from central PRES at all, and instead from uremic encephalopathy (UE). UE typically involves cortical regions, although exclusive basal ganglia and brainstem involvement have been known to occur, which mimics central PRES on FLAIR MRI.^6^ Although the pathophysiological changes between both PRES and UE are not fully understood (purposed autoregulation failure in the former, and toxin mediated in the latter), both result in breakdown of the blood‐brain barrier and subsequent vasogenic edema noted on FLAIR MRI as hyperintense signaling, without DWI findings.^6^ Although both conditions are known to improve with hemodialysis (as a last resort if medical therapy is unsuccessful at controlling blood pressure in PRES patients), we believe that the major contributing factor causing pan‐pontine hyperintense signaling was due to hypertension, with urea most likely playing an exacerbation part. Although her creatinine and BUN were both elevated on admission (3.2 and 52, respectively), our patient was never acidotic, she did not display signs of asterixis at any point, and cortical regions typical of UE were not involved, factors which lead us to believe the culprit was high blood pressure. Additionally, all reports of patients with central PRES to our knowledge present with hypertension (with or without other risk factors such as pre‐eclamptic features, uremia, or immunomodulator use), which is not always the case of UE patients. Although we believe hypertension to be the major cause of central PRES in our patient, we recognize that serious consideration of UE is justified in encephalopathic CKD patients, especially as an alternative diagnosis if blood pressure control fails to improve patients clinically.

To our knowledge, cases of central variant PRES with the entirety of the pons affected by vasogenic edema with associated partial basal ganglia, thalami, cerebellum, and midbrain involvement have not yet been reported. The Gao et al study in 2012, described a patient with central PRES who had a very similar prevention to ours, with a history of chronic kidney disease (CKD) and hypertension, however vasogenic involvement was confined to the pons alone.^7^ In their study, Gao et al made reference to 6 additional case reports of patients with CKD presenting with PRES, all with pontine and/or brainstem involvement, and one with exclusive pontine vasogenic changes. Additionally, all five of the central variant patients in the McKinney study had partial pontine involvement, two of which had recently undergone kidney transplant (on cyclosporine) due to CKD.^2^ At this point, it is reasonable to hypothesize that the pons may perhaps be the most vulnerable region of vasogenic edematous change in patients with central PRES, and maybe even more so in patients with associated underlaying CKD due to albuminuria and/or an increased tendency for electrolyte imbalance. Due to the vulnerability of the pons in this setting, the fact that our patient waited an entire week before presenting to the ED may be the reason why the pons was so severely affected in comparison with the basal ganaglia, thalami, cerebellum, and midbrain. Although the patient from the Gao study also suffered from CKD,^6^ the fact that he presented a day after the onset of symptoms may account for why only the pons was affected, and additional involvement of other areas typical for central PRES may have occurred if he had waited. Further studies to determine whether or not CKD is significantly associated with an increased risk of pontine involvement in patients presenting with central PRES are warranted.

In response to Lee et al's 2018 report concerning albuminocytologic findings within the cerebral spinal fluid (CSF) of a central PRES patient, ours had a slightly higher than normal protein level of 46, however the concentration of albumin within the CSF was measured at 22 (within normal range). Lee et al (including two other small studies^8^, ^9^) made a case that high albumin within the CSF may be the most common profile in PRES patients, but not necessary required for diagnosis. The involvement of central areas of the brain in PRES and increased albumin in the CSF may be due to the heightened permeability of small vessels in this area, and therefore a greater amount of protein leakage into the extravascular space under hypertensive conditions.^3^ Their patient had a confirmed case of central variant PRES with an albumin profile of 63.8 mg/dL and total protein level of 102.6 mg/dL within the CSF. Although again, an albuminocytologic CSF may not be required for diagnosis, as there have been cases (including ours) of central PRES which have not displayed this findings.^5^, ^7^ Whether or not this correlation is significantly associated with central variants PRES is limited however because so few studies of CSF have been reported, let alone their CSF profiled during their hospital stay. Continued reports of central PRES that make a point of reporting CSF findings may allow for future meta‐analysis, and better objective data as to whether high albumin in the CSF is a true positive indicator of central variant PRES.

## 4 CONCLUSION

In conclusion, posterior reversible encephalopathy syndrome (PRES) is a clinicoradiologic syndrome characterized by edematous changes within the brain parenchyma, likely secondary to conditions such as hypertension, immunosuppressive medications, eclampsia/pre‐eclampsia, and antineoplastics. Central variant PRES, found in roughly 4% of cases, spares subcortical white matter as well as parieto‐occipital regions (areas affected by classical PRES), and instead involves deeper structures such as the basal ganglia, the thalami, cerebellum, and pons, the latter of which may be the most vulnerable. Because central PRES is so rare, and because presentation on MRI can mimic many other forms of pathology, cases of central PRES should continue to be reported, as it pulls this variant into common knowledge for radiologists and neurologists, in the hopes of implementing appropriate management in a timely fashion. We recommend CSF analysis be included in future case reports of central variant PRES, with the aim of perhaps finding a signature CSF profile as a means of supporting a definitive diagnosis.

## CONFLICT OF INTEREST

None declared.

## AUTHOR CONTRIBUTION

JMG: contributed as first/primary author. AL: was involved as the supervising physician and author contributor
